# Convention of the Physicians of Ohio

**Published:** 1834

**Authors:** 


### XIII.—Projected Medical Convention of the Physicians of Ohio.

In our last number we announced, that some of our brethren in the capital, had proposed a meeting of our physicians in that town, on the first Monday of January next. A late letter from one of them, informs us, that the indications are in favor of a numerous convention. This is as it should be. The medical conventions and general societies hitherto held, have consisted of delegates only; and the majority of the physicians of the state have never yet come together. Such a meeting would certainly do good to the profession and society, in various ways, and could not fail to prove delightful, at the moment, by the social hilarity and active good feeling it would excite. We venture to indulge the hope, that it will be well attended, and that the first week of January, 1835, will bring to the apothecaries, fewer prescriptions, than usual. Dr. Franklin used to fast one day in the week, that nature, having a holyday, might clean out her streets—the same rule in regard to medicines might be equally useful to the people.

